# Comparison of Semen Quality Before and After Inactivated SARS-CoV-2 Vaccination Among Men in China

**DOI:** 10.1001/jamanetworkopen.2022.30631

**Published:** 2022-09-08

**Authors:** Jialyu Huang, Leizhen Xia, Lifeng Tian, Dingfei Xu, Zheng Fang, Jiaying Lin, Qiongfang Wu

**Affiliations:** 1 Center for Reproductive Medicine, Jiangxi Maternal and Child Health Hospital, Nanchang University School of Medicine, Nanchang, China; 2 Department of Gynecology and Obstetrics, Center for Reproductive Medicine, Tangdu Hospital, Air Force Medical University, Xi’an, China; 3 Department of Assisted Reproduction, Shanghai Ninth People’s Hospital, Shanghai Jiao Tong University School of Medicine, Shanghai, China

## Abstract

This cohort study examines changes in semen quality—including semen volume, sperm concentration, total sperm count, and total and progressive sperm motility—before and after inactivated SARS-CoV-2 vaccination among men in China.

## Introduction

Mass vaccination campaigns have been conducted worldwide to control the COVID-19 pandemic. Despite reassuring safety profiles in clinical trials, vaccine hesitancy remains high among individuals of reproductive age, partially because of fertility concerns.^1^ Recent studies have shown that messenger RNA and viral-vector SARS-CoV-2 vaccinations do not impair sperm parameters among participants.^2,3,4,5^ However, the effects of inactivated SARS-CoV-2 vaccines—the most widely used vaccine type in mainland China—on semen quality have not been assessed. We evaluated changes in semen quality before and after inactivated SARS-CoV-2 vaccination among men in China.

## Methods

This self-controlled, retrospective cohort study was approved by the Reproductive Medicine Ethics Committee of Jiangxi Maternal and Child Health Hospital. Written informed consent was obtained from all patients. Additional details are provided in the eMethods in the Supplement. The study followed the STROBE reporting guideline.

Sperm parameters of patients who had received 2 full doses of either the inactivated BBIBP-CorV (Sinopharm) or CoronaVac (Sinovac) vaccine were collected from June 15, 2021, to April 15, 2022, and compared with their previous semen analysis data within 1 year before vaccination. Vaccination status was ascertained with official immunization records in national and local mobile applets for each patient. Experienced technicians performed all semen collection, handling, and analysis according to World Health Organization laboratory manual procedures.^6^ The prevaccination and postvaccination periods were compared using the paired *t*, Wilcoxon signed-rank, or McNemar χ^2^ test, as appropriate. The postvaccination groups (≤90 and >90 days) were compared using the unpaired *t*, Wilcoxon rank-sum, Pearson χ^2^, or Fisher exact test, as appropriate. *P* < .05 (2-tailed) was considered statistically significant. Statistical analysis was conducted using SAS version 9.4 (SAS Institute Inc).

## Results

The final cohort consisted of 128 men, with a median (IQR) age of 31.0 (29.0-35.0) years and a median body mass index (calculated as weight in kilograms divided by height in meters squared) of 24.2 (22.5-26.2). Only 2 men (1.6%) reported current alcohol consumption; 43 (33.6%) were current smokers. The incidence of hypertension, diabetes, and dyslipidemia was 9.4%, 3.1%, and 50.8%, respectively. Participants reported a median (IQR) sexual activity rate of 1 (1-2) time per week. The median (IQR) serum testosterone level was 386.0 (298.9-521.5) ng/dL. For postvaccination semen analyses, samples were obtained at a median (IQR) of 87.5 (52.0-137.5) days after the second vaccine dose.

Semen quality before and after inactivated SARS-CoV-2 vaccination is summarized in the Table. All parameters were similar between the prevaccination and postvaccination periods, including semen volume, sperm concentration, total sperm count, and total and progressive sperm motility. Given the cycle span of sperm development, vaccinated patients were further subdivided into 2 groups based on the postvaccination interval for semen analysis (≤90 and >90 days); no significant differences were observed.

**Table.** Semen Parameters Before and After Inactivated SARS-CoV-2 Vaccination Among Men in China

Parameter	Prevaccination (n = 128)	Postvaccination			*P* value	
		Total (n = 128)	≤90 d (n = 66)	>90 d (n = 62)	Prevaccination vs postvaccination^a^	≤90 vs >90 d^b^
Semen volume, mean (SD), mL	2.8 (1.2)	2.9 (1.3)	2.8 (2.0-3.5)	2.7 (2.0-3.6)	.44	.86
Sperm concentration, median (IQR), million/mL	39.0 (19.5-60.4)	42.0 (25.2-62.7)	39.3 (19.1-56.9)	45.9 (26.7-63.8)	.29	.12
Total sperm count, median (IQR), million	107.1 (53.3-169.2)	115.5 (50.2-185.9)	107.8 (46.1-164.7)	132.0 (66.9-224.9)	.55	.09
Total motility, mean (SD), %	37.1 (18.9)	37.9 (18.7)	36.1 (19.4)	39.8 (18.0)	.46	.26
Progressive motility, mean (SD), %	30.3 (15.9)	28.6 (16.0)	27.8 (13.7-40.9)	30.0 (19.5-40.7)	.10	.35

^a^ The prevaccination vs postvaccination comparison used the paired *t* or Wilcoxon signed-rank test.

^b^ The postvaccination interval comparison (≤90 vs >90 days) used the unpaired *t* test or Wilcoxon rank-sum test.

To address clinical relevance, sperm parameters were dichotomized as below or above the World Health Organization lower reference limits. Consistently, all outcomes were comparable before and after vaccine exposure (Figure). Of the 128 men in the study, 28 were oligospermic at baseline, 24 remained oligospermic during follow-up (21.1% vs 18.8%; *P* = .65), and none became azoospermic.

**Figure.**
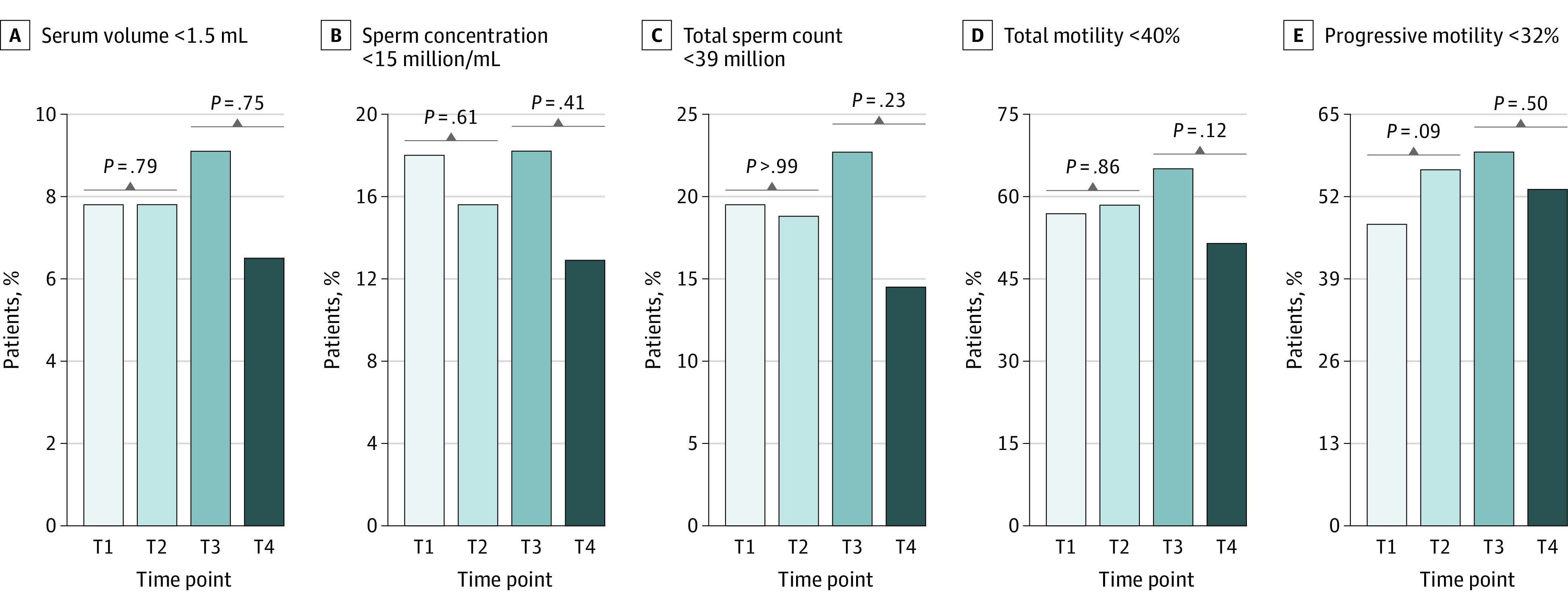
Outcomes of Semen Parameters Below the World Health Organization Lower Reference Limits Before and After Inactivated SARS-CoV-2 Vaccination Among Men in China A, Semen volume below 1.5 mL. B, Sperm concentration below 15 million/mL. C, Total sperm count below 39 million. D, Total motility below 40%. E, Progressive motility below 32%. The prevaccination and postvaccination periods were compared using the McNemar χ^2^ test. The postvaccination groups (≤90 and >90 days) were compared using the Pearson χ^2^ or Fisher exact test, as appropriate. T1 and T2 represent the prevaccination and postvaccination periods, whereas T3 and T4 represent the postvaccination interval (≤90 and >90 days), respectively.

## Discussion

The findings of this cohort study suggest that inactivated SARS-CoV-2 vaccination had no detrimental effect on sperm numbers and motility among men in China. These findings contribute to increasing data regarding the reproductive safety of SARS-CoV-2 vaccines and can be reassuring for vaccinated male patients who are planning a pregnancy.

The self-controlled study design has the strength of being unaffected by between-person confounding but is weakened by unadjusted time-associated covariates (eg, the time between prevaccination and postvaccination periods). Other study limitations include potential selection bias owing to the retrospective design, the heterogeneous population, the small cohort size, inconsecutive and discordant timing of semen analyses during follow-up, and the lack of outcomes such as sperm morphology and DNA fragmentation index. Future large prospective studies are needed to confirm our findings.

## Notes

Supplement.
eMethods

eReferences
