# Supplementary material for: Comparison of Semen Quality Before and After Inactivated SARS-CoV-2 Vaccination Among Men in China
Source: JAMA Netw Open. 2022 Sep 8;5(9):e2230631. doi: 10.1001/jamanetworkopen.2022.30631 (PMC9459660; doi:10.1001/jamanetworkopen.2022.30631)
Supplement: Supplement. — eMethods eReferences [file jamanetwopen-e2230631-s001.pdf]

Supplementary Online Content

Huang J, Xia L, Tian L, et al. Comparison of semen quality before and after inactivated SARS-CoV-2 vaccination among men in China. *JAMA Netw Open*. 2022;5(9):e2230631. doi:10.1001/jamanetworkopen.2022.30631

eMethods

eReferences

This supplementary material has been provided by the authors to give readers additional information about their work.

© 2022 Huang J et al. *JAMA Network Open*.

eMethods

This retrospective cohort study followed the Strengthening the Reporting of Observational Studies in Epidemiology (STROBE) reporting guideline. In total, 4500 semen analyses were performed in 3358 men attending our center during the study period. Among these subjects, 2323 were fully vaccinated with two doses of inactivated COVID-19 vaccines, while the others were unvaccinated (*n*=800), partially vaccinated (*n*=168), or vaccinated with viral-vector (*n*=30) or protein-based (*n*=121) vaccines. Of the fully vaccinated individuals, 146 had undergone an earlier spermogram within one year before vaccination for fertility evaluation and were screened for eligibility. Based on the criteria determined *a priori*, we further excluded three males with pre-vaccination azoospermia, two with chromosomal abnormality, eight with medical treatment for sperm quality improvement, five with missing data in the electronic medical record, and none with prior SARS-CoV-2 infection. The remaining 128 men were included for the final analysis. In conformity with the 2016 Chinese guideline for the management of dyslipidemia in adults,[1] dyslipidemia was defined as serum triglycerides ≥2.3 mmol/L (200 mg/dL), and/or total cholesterol ≥6.2 mmol/L (240 mg/dL), and/or high-density lipoprotein cholesterol <1.0 mmol/L (40 mg/dL), and/or low-density lipoprotein cholesterol ≥4.1 mmol/L (160 mg/dL). According to the 5th edition of World Health Organization laboratory manual,[2] oligospermia was defined as sperm concentration <15 million/mL and/or total sperm count <39 million. For statistical analysis, continuous variables were presented as mean with standard deviation or median with interquartile range (IQR), and compared by t-test (paired or unpaired) or Wilcoxon test (signed-rank or rank-sum) based on normality and independence of samples. The percentages of categorical variables were compared by χ^2 test (Pearson or McNemar) or Fisher's exact test when appropriate.

© 2022 Huang J et al. *JAMA Network Open*.

eReferences

1. Joint Committee Issued Chinese Guideline for the Management of Dyslipidemia in Adults. 2016 Chinese guideline for the management of dyslipidemia in adults [In Chinese]. *Zhonghua Xin Xue Guan Bing Za Zhi*. 2016;44(10):833-853. doi:10.3760/cma.j.issn.0253-3758.2016.10.005
2. World Health Organization. *WHO Laboratory Manual for the Examination and Processing of Human Semen*. 5th ed. Geneva, Switzerland: World Health Organization Department of Reproductive Health and Research; 2010.

© 2022 Huang J et al. *JAMA Network Open*.
